# Sequencing and Genomic Analysis of Sorghum DNA Introgression Variant Line R21 and Recipient Rice Jin Hui 1 Revealed Repetitive Element Variation

**DOI:** 10.3390/ijms231911864

**Published:** 2022-10-06

**Authors:** Ting Zhang, Xiaodong Li, Zijun Zhao, Renhong Wu, Zhenglin Yang, Guanghua He

**Affiliations:** 1 College of Agronomy and Biotechnology, Southwest University, Chongqing 400716, China; 2 Academy of Agricultural Sciences, Southwest University, Chongqing 400716, China

**Keywords:** rice (*Oryza sativa L*.), sorghum *(Sorghum bicolor* (L.) Moench), distant species, genome, heat tolerance, new germplasm

## Abstract

Transferring the genome of distant species to crops is an efficient way to create new germplasms. However, the molecular mechanisms involved are unclear. In this study, a new rice restorer line R21 with heat tolerance was created by introgressing the genomic DNA of sorghum into the recipient restorer line Jin Hui 1. Assembly of rice R21 and Jin Hui 1 genomes was performed using PacBio sequencing technology. Comparative genome analysis and coverage statistics showed that the repetitive sequence atr0026 was a candidate introgression fragment of sorghum DNA. Sequence similarity analysis revealed that atr0026 was distributed at different copy numbers on the telomeric position of chromosomes 9 or 10 in R21, Jin Hui 1, and several rice varieties, indicating that the repetitive sequence from sorghum was highly conserved in rice. The repeat annotation in Gramineae indicated that ribosomal DNA loci that existed in atr0026 may be cause a rearrangement of chromosomes 9 and 10 of the R21 genome, resulting in a copy number variation at the 5′ end of it. Our study lays the foundation for further elucidation of the molecular mechanisms underlying the heat tolerance of sorghum DNA introgression variant line R21, which is of great significance for guiding crop genetic breeding.

## 1. Introduction

Rice (*Oryza sativa L.*) is a major food crop, feeding more than half of the world’s population [1]. As the global population continues to grow, the demand for rice is also increasing. In recent years, global warming has intensified, and high temperatures severely constrain rice yield, posing a severe threat to world food production and security [2,3]. Therefore, the creation of heat-tolerant rice germplasm resources has become a major scientific problem that needs to be solved urgently at present.

Distant cross is one of the most common methods of conventional breeding to create new germplasm. Transferring the genomes of distant species to varieties of recipient plants can generate a wealth of variant material, thus achieving a comprehensive improvement of agronomic traits in the recipient [4,5,6]. Currently, molecular breeding for the direct introduction of genomic DNA from distantly related species into different rice recipients has been widely used [7]. YeWei B is a variant line obtained by introgressing the genomic DNA of wild rice of *O. minuta* into the hybrid rice maintainer line V20B, which showed a narrowed and reduced leaf angle of the top three leaves and improved seed setting rate and seed quality [8]. Genome sequencing of YeWei B showed that 21 of the 28 selected genes associated with differential traits had variants, including 208 single nucleotide polymorphisms (SNPs) and 98 insertion-deletion (InDel) variants [7]. Ma et al. introduced the maize genomic DNA into recipient rice cells by distant molecule hybridization and obtained 18 rice variant line materials with significantly altered phenotypes such as plant height, purplish stems and leaf sheaths, and aerial roots. A total of 308 base mutations were found in these variant lines, and more than half of them were transitions [9]. *ERV1* is a variant line with significantly improved yield-related traits, which is obtained by introgressing genomic DNA of *Oryza eichingeri* into the recipient rice *RH78* [10]. Liu et al. used the pollen-tube pathway to introgress *Fargesia spathacea* Franch DNA into the rice. The obtained variant line T1 had a reduced plant height and increased 1000-grain weight, and T2 had an increased number of effective panicles and grains per panicle but a reduced panicle length [11]. Thus, transferring genomes of distance species has been widely used in the molecular improvement of crops, and it has created a large number of new germplasms and varieties of crops with significantly improved traits [8,12,13,14]. However, the molecular mechanism associated with this process is still unclear.

In this study, the genomic DNA of the high photosynthetic efficiency dryland crop sorghum (*Sorghum bicolor* (L.) Moench) was introgressed into the rice recipient restorer line Jin Hui 1 to create a new rice restorer line R21, which showed heat tolerance at the flowering stage [15,16]. We used PacBio sequencing technology for the de novo assembly of genomes of R21 and Jin Hui 1, performed comparative analysis between genomes with *Japonica*, and identified abundant genomic differences. The whole-genome coverage statistics showed that the potential introgression fragment of Sorghum DNA was located within 45.4–45.6 Mb of chromosome 5 of Sorghum. Further comparative analysis demonstrated that a 5.8 kb sequence among 200 kb intervals can be screened as a candidate introgression fragment due to its highest homology with the R21 genome. The fragment annotated as a repeat sequence (atr0026) in sorghum was distributed at different copy numbers on the telomeric position of chromosomes 9 or 10 in R21, Jin Hui 1, and several rice varieties, indicating that this repeat sequence from sorghum is highly conserved in rice. The repeat annotation from Gramineae indicated that the repeat atr0026 on sorghum contains a conservative 18s ribosomal DNA loci, and its introgression may be cause the genome rearrangement of chromosomes 9 and 10 of the R21, resulting in the presence of CNV at its 5′ end, which may be responsible for the heat tolerance of R21. These results lay the foundation for an in-depth analysis of the molecular mechanism related to germplasm innovation through exogenous gene introgression, which is of great theoretical and practical significance for guiding crop genetic breeding.

## 2. Results

### 2.1. Comparative Analysis of Genomes of R21 and Jin Hui 1

**Figure 1:**
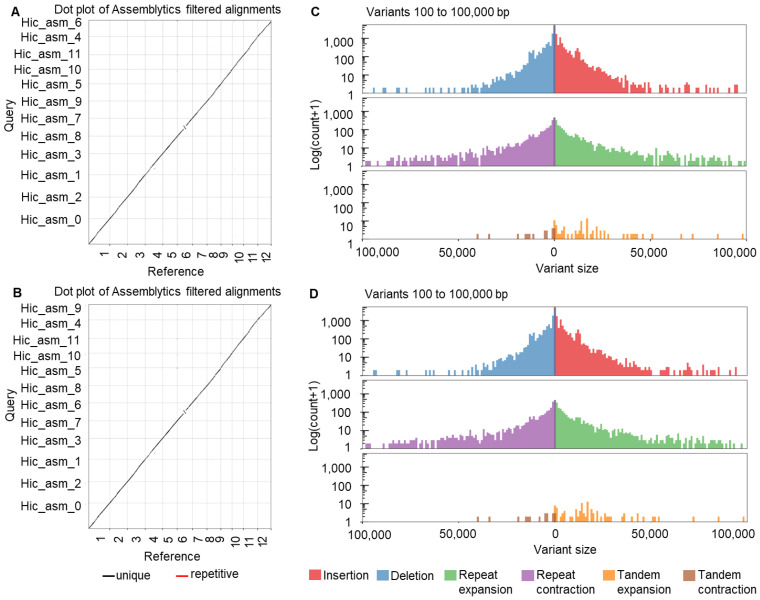
Comparative genome analysis of R21 and Jin Hui 1. (**A**,**B**) Comparative analysis of assembled genomes with rice reference genome IRGSP-1.0 of R21 (**A**) and Jin Hui 1 (**B**); (**C**,**D**) Variation analysis of assembled genomes of R21 (**C**) and Jin Hui 1 (**D**).

The rice genome IRGSP-1.0 (Oryza_sativa—Ensembl Genomes 53) was used as the reference genome. The assembled genomes of R21 and Jin Hui 1 were aligned against reference genome respectively by Minimap2, and presence/ absence variations (PAVs) were identified by Assemblytics software. Based on the alignment results, we re-corrected the chromosome numbering of the assembled genomes of R21 and Jin Hui 1 firstly (Figure 1A,B) (Supplemental Table S1), and analyzed the PAV variations, respectively (Figure 1C,D). The comparative analysis between genomes showed abundant genomic variations, among which 11,880 insertions, 9893 deletions, 83 tandem expansions, 15 tandem contractions, 2159 repeat expansions and 2126 repeat contractions were found in R21; 11,953 insertions, 10,010 deletions, 78 tandem expansions, 17 tandem contractions, 2189 repeat expansions and 2138 repeat contractions were found in Jin Hui 1 (Figure 1C,D).

### 2.2. Identification of Insertion/Homologous Fragments in Sorghum

**Figure 2:**
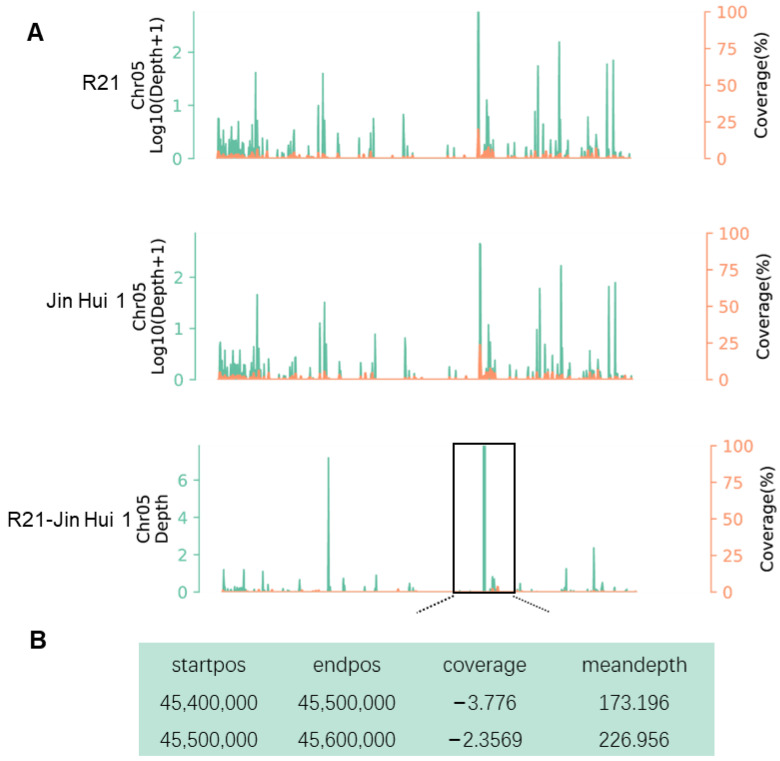
Identification of insertions/homologous fragments in sorghum. (**A**) Distribution of depth of coverage of chromosome 5 between R21 and Jin Hui 1; (**B**) Region of significant difference in depth of coverage between R21 and Jin Hui 1 (top 1%). Green represents depth, Orange represents coverage.

Based on the PacBio sequencing data of R21 and Jin Hui 1, we mapped HiFi reads to the sorghum genome and found that the mapping rate of R21 was 20.4%, which was 0.4% higher than that of Jin Hui 1 (20%) (Figure 2A). After that, we calculated the depth of coverage of HiFi reads on the sorghum genome and the depth of difference, and we identified a total of three candidate intervals (top 1%) on sorghum chromosome 5, in which the depth of difference in coverage of HiFi reads on 45.4–45.5 Mb and 45.5–45.6 Mb reached 170 (Figure 2B). Since the two intervals were adjacent to each other, we combined them into one interval of 200 kb for the subsequent analysis (Figure 2B).

### 2.3. Collinearity Analysis of Insertion/Homologous Fragments of Sorghum with R21 and Jin Hui 1 Genomes

**Figure 3:**
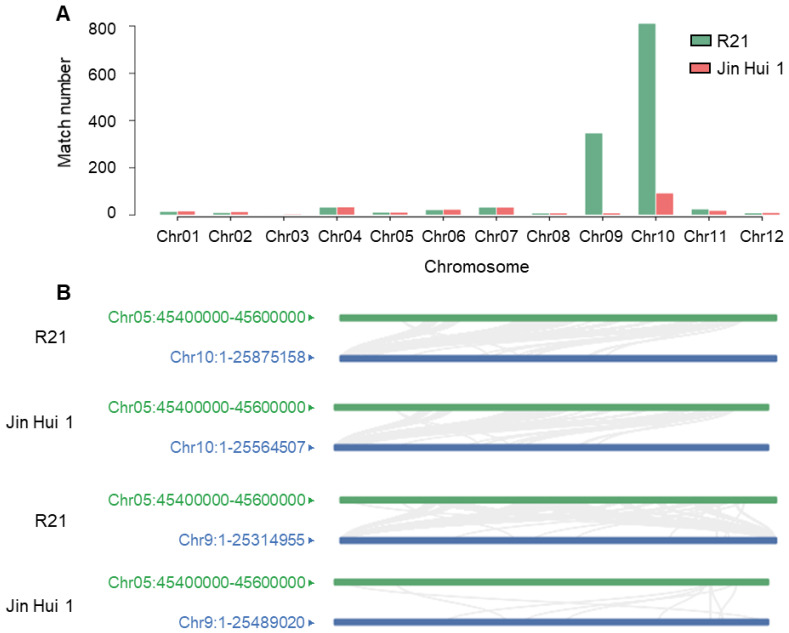
Collinearity analysis of sorghum insertions/homologous fragments with R21 and Jin Hui 1 genomes. (**A**) The alignment results of sorghum candidate analysis sequences blast against two rice genomes; (**B**) The collinearity results of sorghum candidate analysis sequences with chromosomes 9 and 10 of R21 and Jin Hui 1 genomes. Green labels represent the 45.4–45.6 Mb interval of chromosome 5 in sorghum, blue labels represent the full length of chromosomes 9 and 10 in rice.

We extracted 200 kb DNA sequences from the sorghum genome on the 45.4–45.6 Mb interval on chromosome 5 and used BLAST to align to the assembled R21 and Jin Hui 1 genomes, respectively. Based on the alignment results, we found that this candidate sequence of sorghum differed significantly in the number of matches on chromosomes 9 and 10 on both R21 and Jin Hui 1 genomes (Figure 3A). Second, despite the difference in the number of matches, homologous sequences were indeed found on both R21 and Jin Hui 1 genomes, suggesting that this candidate sequence of sorghum itself is likely to be highly homologous to rice (Supplemental Figures S1 and S2). Finally, by analyzing the alignment information (including length, identity, and coverage) within all match regions, we hypothesized that the length of this introgressed sequence in sorghum is approximately 5 kb, mainly affecting the telomeric region of R21 genome chromosomes 9 or 10 (Figure 3B). In summary, the introgression of the sorghum homologous sequence resulted in a difference in coverage depth between R21 and Jin Hui 1; the size of the sorghum homologous introgressed sequence was about 5.8 kb.

### 2.4. Comparative Analysis of Sorghum Insertion/Homologous Fragment, R21, Jin Hui 1 and Japonica Genomes

**Figure 4:**
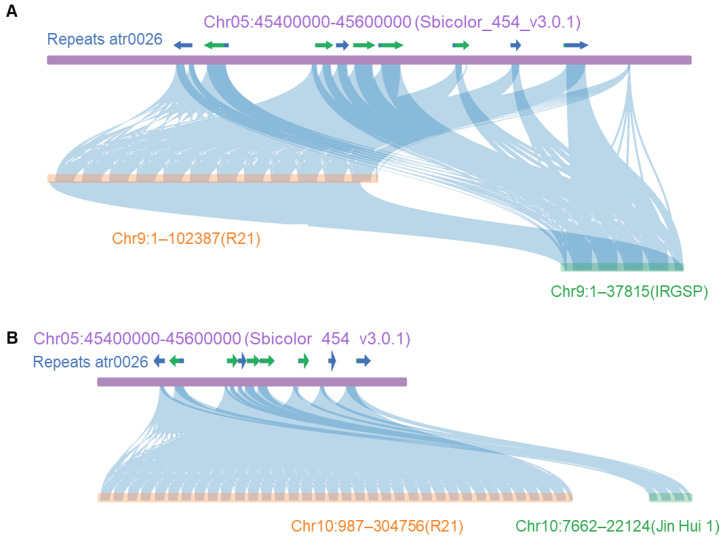
Collinearity analysis of sorghum insertion sequences. (**A**) Collinearity analysis between Sorghum insertion sequence and chromosome 9 of rice (from top to bottom: sorghum Chr05: 45.4–45.6 Mb fragment, repeat annotation atr0026, chromosome 9 segment of R21 genome, chromosome 9 segment of IRGSP genome); (**B**) Collinearity analysis between sorghum insertion sequence and chromosome 10 of rice (from top to bottom: sorghum Chr05: 45.4–45.6 Mb fragment, repeat annotation atr0026, chromosome 10 segment of R21 genome, partial fragment of chromosome 10 in Jin Hui 1 genome). Consistency between sequences was greater than 90%. Arrows represent repeats atr0026, and blue and green are used to distinguish the atr0026 overlapped.

We extracted a 5.8 kb fragment from the candidate region of sorghum (Chr05: 45.4–45.6 Mb), which has the highest similarity with the rice genome as the candidate insertion sequence, and then compared with R21, Jin Hui 1 and IRGSP of the Japonica genome by BLAST. First of all, according to the results of sorghum genome repeat sequence annotation, we found that the 5.8 kb insertion sequence is a repeat sequence atr0026, and it was repeatedly annotated in the Chr05: 45.4–45.6 Mb interval (Figure 4A,B). Secondly, based on the previously calculated reads coverage rate of 1 kb in Chr05: 45.4–45.6 Mb of sorghum, we found that the region with reads coverage is almost consistent with the position distribution of repeat atr0026 (Figure 4A,B). To confirm the presence of the repeat atr0026 on sorghum in other species, we used the BLAST function (with parameters e-value 1 × 10^−5^ and word length 11) on RGAP (http://rice.uga.edu/, accessed on 20 September 2022) [17] to match the sequence to the Gramineae Repeat Database. According to the results, there is a repeat sequence belonging to ribosomal DNA in wheat (*Triticum aestivum*), rice (*Oryza sativa*), maize (*Zea mays*), and Foxtail Barley (*Hordeum jubatum*), which can be matched to the front end of the repeat atr0026. Among them, there is another repeat sequence in rice and wheat that can be matched to the back end of the repeat atr0026. This result indicated that the repeat atr0026 on sorghum contains a conservative 18s ribosomal DNA loci, which exists in Gramineae species (Supplemental Table S2) (Supplemental Figure S3).

Finally, based on the results of sequence alignment, we found that the 5.8 kb repeat sequence atr0026 in sorghum has multiple high homology alignments on the chromosome 9 of R21 and IRGSP genomes (sequence consistency is greater than 90%), and the high homology alignment frequency (12 times) on the Chr9 chromosome of the R21 genome is much higher than that on the Chr9 chromosome of the IRGSP genome (4 times). However, no homologous region was found on chromosome 9 of the Jin Hui 1 genome (Figure 4A). In addition, in the reference genome of Japonica, there are two candidate genes, *LOC_Os09g00999* and *LOC_Os09g01000*, with unknown function in the region corresponding to this repeat sequence. At the same time, the sequence also has several high homology alignments on Chr10 chromosomes of R21 and Jin Hui 1 genomes, and the number of high homology alignments on chromosome 10 of R21 genome (35 times) is much higher than that of the Jin Hui 1 genome (4 times). However, no homologous region was found on chromosome 10 of the IRGSP genome (Figure 4B). In summary, the introgression of the sorghum sequence may be cause of a repetitive region occurring on chromosome 9 and 10 in R21, resulting in CNV (copy number variation) at the 5′ end, up to 47 times.

### 2.5. Collinearity Analysis between Sorghum Candidate Sequences and Rice Pan Genome

**Figure 5:**
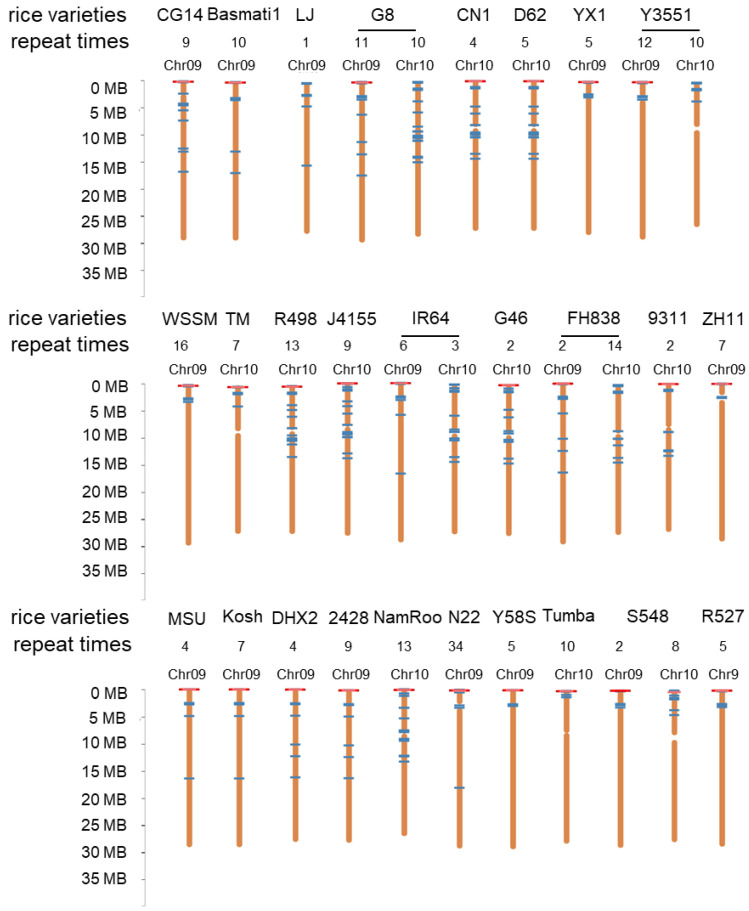
Homology alignment of 5.8 kb sequence from sorghum with 33 rice genomes on chromosomes (over 90% sequence consistency).

To confirm whether the 5.8 kb repetitive sequence of sorghum is homologously conserved in all current rice varieties, we also compared the sequence with 33 rice genomes on the rice pan genome website (http://ricerc.sicau.edu.cn/, accessed on 20 September 2022) by BLAST [18]. Based on the comparison results, we found that this 5.8 kb repeat sequence atr0026 on the sorghum genome is distributed on the genomes of different rice materials, where it is mainly found on chromosome 9 or 10 (Figure 5). According to statistics, this sequence repeats 110 and 154 times on chromosomes 9 and 10 in 27 rice varieties, respectively, and there are no homologous sequences in six rice varieties. Among the 27 rice varieties, N22 is a strong heat-tolerant rice variety, which originated from India with high temperature and a high humidity environment [19]. It is worth noting that in N22, the candidate sequence of sorghum appeared 34 times on chromosome 9 (Figure 5).

## 3. Discussion

So far, many examples have shown that the introgression of favorable exogenous genes in rice plays a vital role in improving the yield, quality, and resistance, which is an effective tool for the innovation of rice germplasm resources [20,21,22,23]. Among the distant hybrids of rice, crosses with sorghum have been more intensively and maturely studied. Sorghum is a C4 plant with high photosynthetic efficiency, fast growth rate, good photosynthetic products, high adaptable, strong heat tolerance, and stable and high yield [24]. Some excellent characteristics of sorghum are transferred to rice through hybridization, and new rice varieties with high yield and high quality can be bred. *SbSGL* encodes a DUF1645 superfamily protein in sorghum, and its transfer to rice significantly increased the size and number of glume cells and post-fertilization filling rate, which in turn led to a highly significant increase in grain length and 1000-grain weight [20]. Qin et al. introgressed sorghum cDNA into the rice and more than 92% of the 965 transgenic lines obtained different phenotypic changes, including longer leaves and increased effective spike number [21]. In this study, R21 was a rice variant line obtained by introgressing sorghum DNA into the rice restorer line Jin Hui 1, and the content proline, SOD and POD activities, and MDA of R21 were significantly increased after being subjected to high temperature and drought stress, indicating that R21 has excellent heat tolerance and drought tolerance [15]. Therefore, exogenous gene introgression can significantly improve rice traits and is an effective way of creating new germplasm resources and crop varieties.

The key to hybrid rice breeding is to produce stress-resistant, high-quality, and high combining ability sterile and restorer lines with excellent agronomic traits, which is an essential requirement for assembling widely adapted, highly dominant rice hybrid combinations. R21 is an excellent restorer parental material, which has excellent characteristics, such as strong heat and drought resistance, compact plant shape, stiff stalks, strong resistance to falling, upright leaves, neat panicles, long spikes and high seed-setting rate [15,16]. Many breeders use R21 as the parent material to cultivate hybrid rice varieties. Based on data from the Chinese Rice Data Center [25], seven varieties bred by using R21 passed the examination 13 times. The regional and production test results showed that the average yield of the seven varieties out of the the 13 examined increased by 5.33% and 8.88%, and the highest yield increases were 10.26% and 16.26%, which were higher than the varieties approved for the same period. Among them, Fu You 1, bred from the combination of male sterile line II-32A and R21, is the first national approved rice variety selected by an exogenous DNA introgression technique in China. Fu You 1 has passed the national approval of the upper, middle and lower reaches of the Yangtze River, the Wuling Mountains, as well as the approval of Hainan and Vietnam. The average yield increase in Fu You 1 in the regional test and production test is higher than that of the varieties in the same period. From 2003 to 2021, there were 1181 hybrid Indica rice varieties that were examined and approved by the Ministry of Agriculture in the Yangtze River basin, and only six varieties were certified in three zones simultaneously. Fu You 1 is not only one of them but also ranks first in the average yield increase in regional and production trials (8.35% on average).

Genome sequencing is the most direct and effective method for analyzing the genomic variation. DNA sequencing technology has undergone three technological revolutions [26]. In this study, we found that after the introgression of Sorghum DNA, there are obvious differences between the R21 and Jin Hui 1 genomes. Repeat atr0026 on sorghum contains a conservative 18s ribosomal DNA loci, which exists in Gramineae species. It has been proved that ribosomal DNA is a kind of common characteristic in Oryza and even in Gramineae, but because of the positional variability or transpositional nature of ribosomal DNA, the position of ribosomal DNA is different in different species, and this difference may be determined in the process of evolution [27]. Therefore, we speculate that since atr0026 on sorghum contains ribosomal DNA loci, when it introgressed into the rice genome, it affected the location and number of rDNA of R21 on chromosome 9 and chromosome 10, which in turn led to the fragment highly homologous to atr0026 recurring many times on rice. The repetitive sequence atr0026 is distributed in different degrees in all published rice pan genomes, with 34 occurrences on chromosome 10 of the strongly heat-tolerant variety N22 and up to 47 occurrences on chromosomes 9 and 10 of R21, further suggesting that the number of duplications of the repeat atr0026 is closely associated with the formation of traits in R21.

## 4. Materials and Methods

### 4.1. Plant Materials

The two plant materials used in this study were the japonica restorer line Jin Hui 1 and the variant line R21. R21 was obtained by using Jin Hui 1 as the acceptor and sorghum DNA as the donor. The sorghum DNA is injected into the young panicle of Jin Hui 1 by the pollen-tube pathway at the meiotic stage. R21 has excellent heat tolerance. After five years and 13 generations of directional selection and comparative identification of the variant line, the traits can be inherited stably [16].

### 4.2. Rice Genome Sequencing and Assembly

In this study, PacBio sequencing was completed in Novogene Bioinformatics Technology Co., Ltd. High molecular weight DNA was first extracted from the seedlings of R21 and Jin Hui 1. A SMRT bell library was constructed as previously described [28] and sequenced on the PacBio Sequel II platform (Pacific Biosciences). The PacBio HiFi reads obtained by sequencing were assembled de novo using Hifiasm software [29]. The integrity of the assembled genome was assessed using BUSCO [30] and GEGMA software [31].

### 4.3. Comparative Analysis and Variant Identification of Rice Genome

The two rice assembled genomes of R21 and Jin Hui 1 were compared with the rice genome IRGSP-1.0 (Oryza_sativa—Ensembl Genomes 53) using Minimap2 software [32], and PAV variants were identified by Assemblytics software [33].

### 4.4. Screening and Collinearity Analysis of Homologous Sequences in Sorghum

The HiFi reads obtained by PacBio third-generation sequencing were stored in BAM format files. Therefore, we first converted the format of files from BAM to FASTQ by SAMtools software [34], and then, the HiFi reads of the two rice materials were aligned with the sorghum reference genome (https://phytozome-next.jgi.doe.gov/, accessed on 20 September 2022) using Minimap2. After obtaining the comparison results, we used SAMtools to count the coverage and sequencing depth within each 100 kb sequence of the sorghum genome, and we calculated the difference in the coverage depth of the two rice materials within the same sequence.

According to the coverage depth difference region, the coverage depth and coverage rate of each 1 kb sequence were further calculated. Then, the DNA sequence corresponding to this interval was extracted from the sorghum genome as a candidate analysis sequence, which was aligned with the assembled R21 and Jin Hui 1 and the rice pan-genome, respectively, using BLAST to confirm the collinearity of the sequences [35].

### 4.5. Repeat Annotation in Gramineae

The DNA sequence of repeat was submitted to RGAP (http://rice.uga.edu/, accessed on 20 September 2022) for identifying and classifying this repeat in the Gramineae Repeat Database [36] using the BLAST function with an e-value 1 × 10^−5^ and word length of 11.

## 5. Conclusions

In this study, a new rice restorer line R21 with heat tolerance was created by introgressing the genomic DNA of sorghum into the recipient restorer line Jin Hui 1. We used PacBio sequencing technology for the de novo assembly of genomes of R21 and Jin Hui 1. The candidate introgression fragment of sorghum DNA was a repetitive sequence (atr0026), which was distributed at different copy numbers on the telomeric position of chromosomes 9 or 10 in R21, Jin Hui 1, and several rice varieties, indicating that the repetitive sequence from sorghum is highly conserved in rice. The repeat annotation in Gramineaes indicated that ribosomal DNA loci existing in atr0026 may be cause a rearrangement of chromosomes 9 and 10 of the R21 genome, resulting in a CNV at the 5′ end of it. Our study lays the foundation for further elucidation of the molecular mechanisms underlying the heat tolerance of sorghum DNA introgression variant line R21, which is of great significance for guiding crop genetic breeding.

## Data Availability

The raw sequence data have been deposited in the Genome Sequence Archive (GSA) database in BIG Data Center (https://ngdc.cncb.ac.cn/, accessed on 30 September 2023), under accession number PRJCA011922. All other data are available from the corresponding author upon reasonable request.

## Supplementary Materials

The following supporting information can be downloaded at: https://www.mdpi.com/article/10.3390/ijms231911864/s1.
