# Supplementary material for: Sequencing and Genomic Analysis of Sorghum DNA Introgression Variant Line R21 and Recipient Rice Jin Hui 1 Revealed Repetitive Element Variation
Source: Int J Mol Sci. 2022 Oct 6;23(19):11864. doi: 10.3390/ijms231911864 (PMC9570325; doi:10.3390/ijms231911864)
Supplement: Supplementary file 1 [file ijms-23-11864-s001.zip › ijms-1909856-supplementary.pdf]

Supplemental Table S1. The refined chromosome number based on IRGSP-1.0.

Length(bp)	R21	Length(bp)	Jin Hui 1	Length(bp)
43270923	Hic_asm_0	45201676	Hic_asm_0	45199504
35937250	Hic_asm_2	37380711	Hic_asm_2	38037874
36413819	Hic_asm_1	39681045	Hic_asm_1	39462501
35502694	Hic_asm_3	36946273	Hic_asm_3	37200980
29958434	Hic_asm_7	31055467	Hic_asm_8	30861223
31248787	Hic_asm_6	31827323	Hic_asm_7	32026469
29697621	Hic_asm_8	29968225	Hic_asm_9	29984113
28443022	Hic_asm_5	31920571	Hic_asm_5	30568245
23012720	Hic_asm_10	25314955	Hic_asm_10	25489020
23207287	Hic_asm_11	25875158	Hic_asm_11	25564507
29021106	Hic_asm_4	32780025	Hic_asm_4	32677099
27531856	Hic_asm_9	27486733	Hic_asm_6	28727372

Supplemental Table S2. BLAST analysis of repeat atr0026 on Gramineae.

Accession	Description	Hit Score	E value	Num HSPs	Top query coverage	Top identity
ORSiRGRR 00000367	gi|37545144|gb|AY120865.1| Oryza longistaminata small subunit ribosomal RNA gene, partial sequence	1578	0	1	30.89	97.1
TRSiRGRR 00000061	gi|15982656|gb|AY049040.1| Triticum aestivum 18S ribosomal RNA gene, partial sequence	1582	0	1	31.12	96.95
ZRSiRGRR 00000002	gi|1777706|gb|U42796.1|ZMU42796 Zea mays 18S ribosomal RNA gene, partial sequence	1526	0	1	30	96.9
HRSiRGRR 00000017	||gi|471983 471985 471986 gi|6273812|gb|AF168852.1|AF168852 Hordeum jubatum 18S small subunit ribosomal RNA gene, complete sequence	1523	0	1	29.94	96.89
TRSiRGRR 00000030	gi|7209267|emb|AJ272181.1|TAE272181 Triticum aestivum 18S rRNA gene for 18S ribosomal RNA, strain MV-15 ||gi|3402540	1544	0	1	30.39	96.88
ORSiRGRR 00000008	gi|20359|emb|X00755.1|OSRRN17S Rice gene for 17S ribosomal RNA ||gi|3342031 472184 472185 472186 472187 472188 472189 472183 18S small subunit ribosomal RNA gene	1569	0	1	31.2	96.8

ORSiRGRR 00000034	gi|3342031|gb|AF069218.1|AF069218 Oryza sativa 18S small subunit ribosomal RNA gene, complete sequence	1515	0	1	30.08	96.79
ZRSiRGRR 00000004	gi|168608|gb|K02202.1|MZERG17S Maize 17S ribosomal RNA gene and flanks	1481	0	1	31.89	95.48
TRSiRGRR 00000062	gi|15982657|gb|AY049041.1| Triticum aestivum 28S ribosomal RNA gene, partial sequence	2089	0	2	47.95	93.99
ORSiRGRR 00000006	gi|169818|gb|M11585.1|RICRGHA Rice 25S ribosomal RNA gene ||gi|175854 427193 472191 472195 28S rRNA gene gi|169815 2796928 2799921 25S rRNA gene	2426	0	1	58.3	93.42

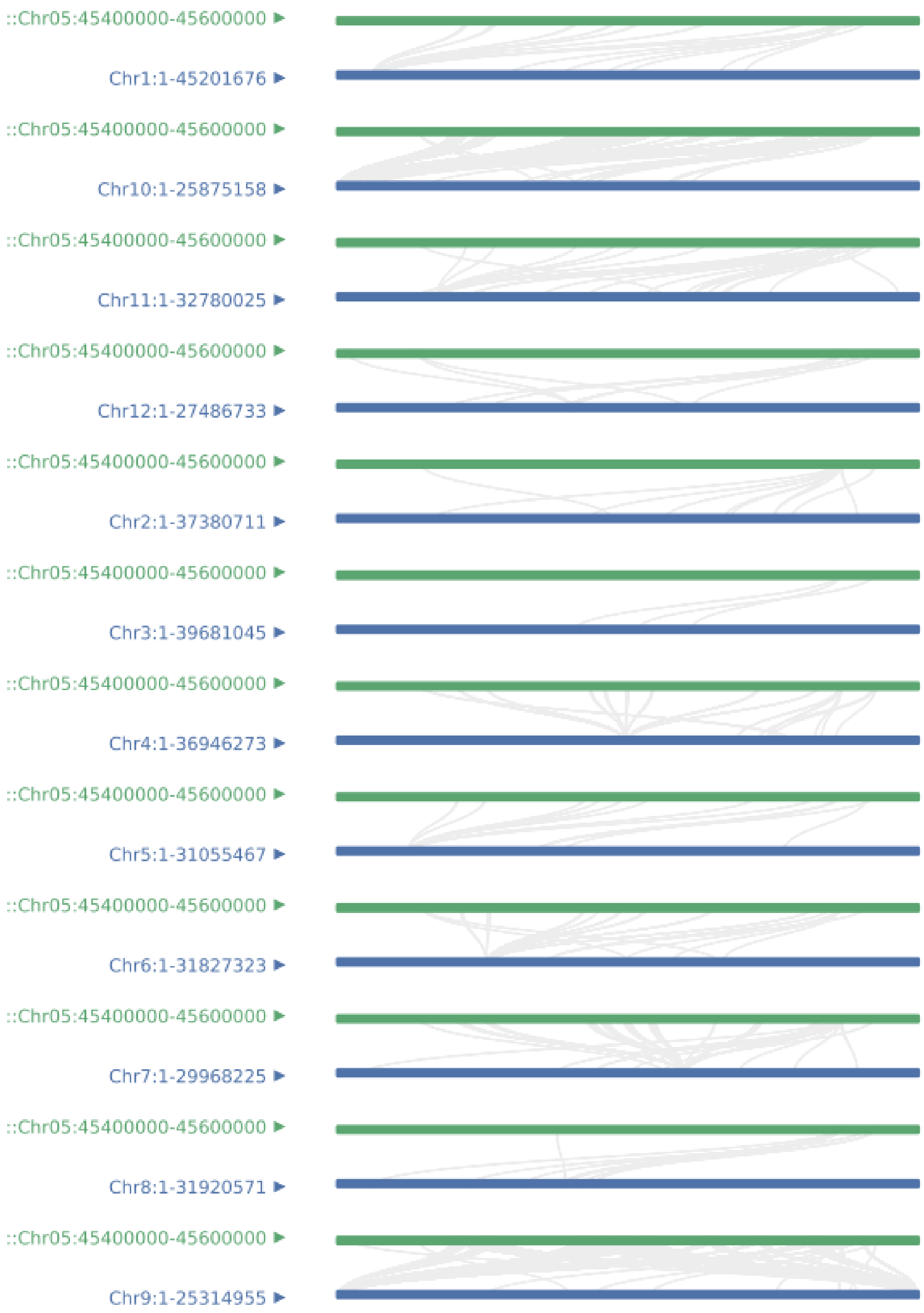

Supplemental Figure S1. Colinearity analysis of sorghum candidate analysis sequences with chromosomes of R21 genome.

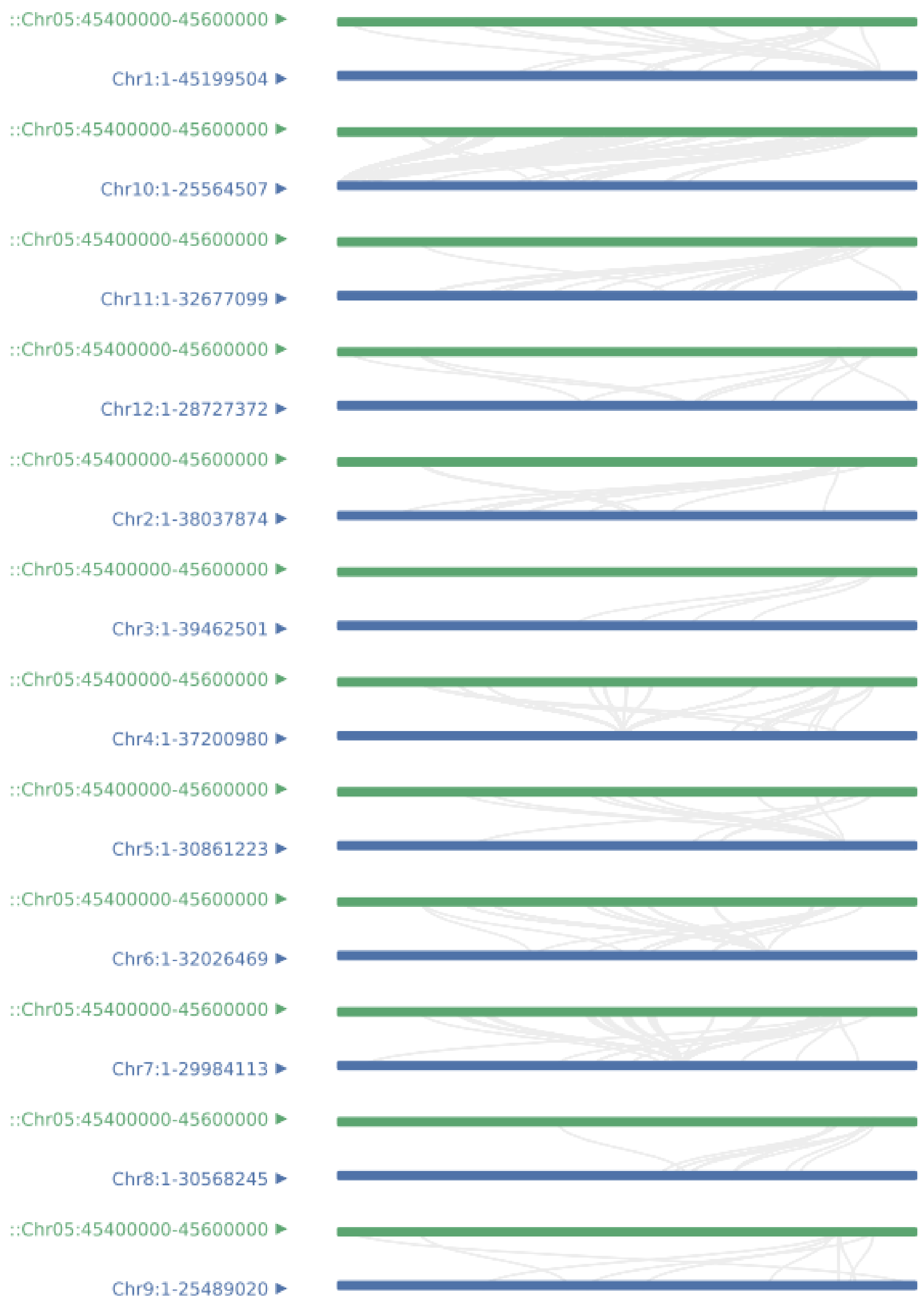

Supplemental Figure S2. Colinearity analysis of sorghum candidate analysis sequences with chromosomes of Jin Hui 1 genome.

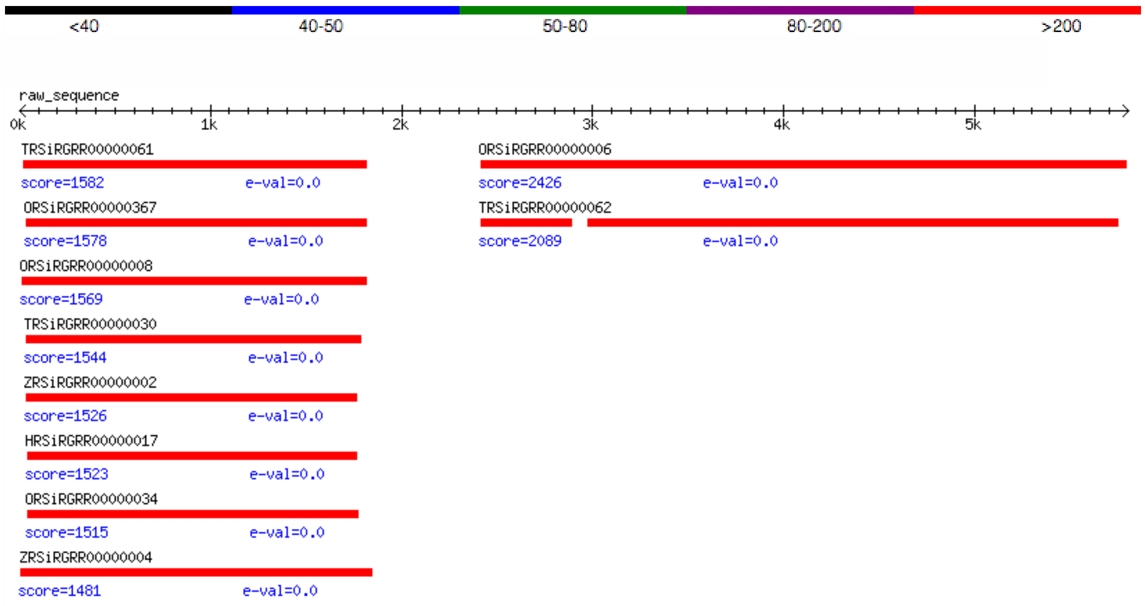

Supplemental Figure S3. BLAST analysis of repeat atr0026 on Gramineae. Legend represents Hit score.
